# Seed Oils as a Hypothesized Contributor to Heart Disease: A Narrative Synthesis

**DOI:** 10.7759/cureus.102012

**Published:** 2026-01-21

**Authors:** Joseph Mercola

**Affiliations:** 1 Independent Researcher, Midwestern University Chicago College of Osteopathic Medicine, Downers Grove, USA

**Keywords:** atherosclerosis, cardiovascular mortality, coronary heart disease, dietary transition, historical epidemiology, linoleic acid, lipid peroxidation, seed oils

## Abstract

Coronary heart disease (CHD) was relatively uncommon in the 19^th^ century, when infectious illnesses dominated mortality, but it rose dramatically in the 20^th^ century in parallel with major dietary shifts, including an increase in linoleic acid (LA), an essential omega-6 polyunsaturated fatty acid (PUFA) abundant in vegetable oils. This review examines whether the rapid, unprecedented rise in consumption of LA-rich industrial seed oils may have played a contributing role in the escalation of CHD. Historical trends in CHD and overall cardiovascular mortality were examined in relation to shifts in dietary fat sources, especially seed oils, and mechanistic studies were reviewed to assess how excessive LA intake could promote atherosclerosis through oxidative stress and inflammation. Multiple lines of evidence were integrated, including early 20^th^-century mortality records, data on dietary fat supply, and findings from experimental studies. Available data indicate that per capita seed oil consumption rose sharply in the early 1900s, preceding the surge in CHD deaths by one to two decades, roughly the time frame needed for atherosclerotic plaques to develop. Soybean oil, in particular, went from virtually no use at the start of the century to a dominant dietary fat by its end, more than doubling the proportion of LA in the food supply and coinciding with a marked rise in LA content within human tissues.

Mechanistic studies further show that LA oxidation can generate reactive aldehydes, such as 4-hydroxynonenal (4-HNE), which have been shown to trigger inflammatory and oxidative pathways. These include activation of the transcription factor NF-κB, which regulates immune signaling, and up-regulation of the protein Bcl-2, which promotes cell survival. These effects can impair endothelial functions central to atherogenesis. While other factors, like cigarette smoking and improved diagnostic tools, also likely contributed to a rise in reported CHD rates, these patterns may not fully account for the magnitude or timing of the mid-century heart disease surge. Taken together, the historical, epidemiologic, and mechanistic evidence suggests that excessive consumption of LA-rich seed oils may have been a significant, under-recognized contributor to the 20th-century CHD epidemic. Reducing the intake of these oils and rebalancing the fatty-acid profile of the diet may therefore be a practical strategy to mitigate CHD risk in modern populations.

## Introduction and background

Coronary heart disease (CHD) contributed only modestly to 19th-century mortality, which was dominated by infectious diseases. Beginning in 1900, however, age-adjusted CHD deaths among U.S. adults ≥ 35 years climbed from 137 to ≈ 450 per 100,000 by 1968. When stroke is included, cardiovascular disease (CVD) mortality peaked at 1,350 per 100,000, a nearly three-fold increase that coincided with major dietary shifts. This increase also occurred in parallel with broader societal changes, including increased lifespan, improved survival from infectious diseases, and a rise in cigarette smoking that may have contributed to the evolving cardiovascular risk landscape. This review explores whether the rapid adoption of industrial seed oils, exceptionally rich in linoleic acid (LA), may have been a critical but under-recognized catalyst of the twentieth-century CHD epidemic.

The aim was to (i) examine temporal trends in CHD and CVD mortality alongside changes in seed-oil consumption; (ii) evaluate the biological plausibility that excess dietary LA may contribute to atherogenesis through oxidative stress and inflammation; and (iii) assess the relative contribution of seed-oil uptake compared with other contemporaneous factors such as smoking prevalence and improved diagnostic tools.

A narrative synthesis approach was used to integrate three primary evidence streams: (1) U.S. national vital-statistics reports (1890-1970) on age-adjusted mortality from coronary heart disease (CHD) and stroke; (2) United States Department of Agriculture (USDA) food-availability tables (1909-1999) detailing per-capita seed-oil supply, with emphasis on soybean oil; and (3) PubMed-indexed, English-language mechanistic studies (through June 2025) examining LA oxidation, lipid peroxides, and vascular inflammation.

Trends in CHD mortality were graphed alongside dietary LA availability, and estimated lag periods between dietary shifts and clinical endpoints were qualitatively compared with the known timeline of atheroma development derived from autopsy and imaging studies. Given the narrative design, this review integrated temporally aligned data without inclusion/exclusion criteria or quantitative meta-analysis.

The per capita seed-oil availability doubled from 2.7 kg to 5.4 kg between 1899 and the early 1910s, preceding the steepest CHD mortality rise by roughly 10-20 years, consistent with the interval required for fatty streaks to progress to occlusive lesions. Soybean oil supply increased from < 0.01 kg in 1909 to > 11 kg in 1999, elevating estimated LA intake from 2.8% to 7.2% of total energy. Concurrently, epidemiologic studies documented a 136% rise in adipose-tissue LA, and experimental research demonstrated that LA oxidation yields cytotoxic aldehydes (e.g., 4-hydroxynonenal) that activate NF-κB, up-regulate Bcl-2, and impair endothelial function, all processes involved in atherogenesis. Although cigarette smoking and wider use of electrocardiography improved detection and added risk, their temporal patterns do not fully account for the magnitude or timing of the mortality surge.

Historical, epidemiological, and mechanistic evidence collectively suggest a strong temporal correlation between the rise in LA-rich industrial seed oil consumption and the 20th-century CHD epidemic. While this dietary shift may have played a significant role, it occurred alongside other major changes in lifestyle and environment, including overall increased lifespan, higher rates of cigarette smoking, greater consumption of processed carbohydrates and refined sugars, and more sedentary living. Reducing dietary intake of seed oils and restoring a balanced fatty-acid profile in the food supply may nonetheless offer a practical, population-level approach to mitigating ongoing CHD risk in the 21st century.

Historical context of coronary heart disease (1850-1900)

In contemporary times, cardiovascular diseases constitute the predominant cause of mortality in the United States, claiming approximately 655,000 lives annually and posing a substantial public health challenge [1]. By contrast, in the mid-19th century, heart disease played a minor role in the landscape of American mortality, overshadowed by the prevalence of infectious diseases such as tuberculosis and pneumonia [2,3]. These infections dominated morbidity and mortality statistics and directed clinical and therapeutic priorities. Before 1900, heart disease contributed to fewer than 100 deaths per 100,000 individuals, representing roughly 8% of total mortality, based on retrospective analyses from early 20th-century data [2,3]. The cardiovascular system, though critical to human physiology, rarely emerged as a focal point of medical concern in this era.

Coronary heart disease (CHD), defined by the narrowing of coronary arteries and subsequent myocardial ischemia, was rarely encountered in 19th-century clinical practice [4]. Myocardial infarctions (MI), in which the heart muscle suffers irreversible damage from sustained oxygen deprivation, were infrequently documented, likely reflecting both their genuinely low incidence and the diagnostic limitations of the time [5]. The vascular endothelium, now recognized as a dynamic regulator of blood flow and the earliest site of atherosclerotic changes, had not yet been conceptualized as such, and no cohesive framework for identifying or understanding CHD existed [6,7]. As a result, CHD remained largely invisible in the medical literature and mortality records of the era, making its historical rarity difficult to interpret with certainty.

Clinical observations underscore the diagnostic obscurity surrounding coronary CHD. In Austin Flint’s hospital casebooks (1870-1890), only seven instances of angina pectoris, a symptomatic hallmark of CHD involving chest pain due to myocardial oxygen deficit, were recorded among 150 patients with significant heart disease, yielding a prevalence of approximately 4.7% [8]. Flint concluded that “angina pectoris is a rare affection,” contrasting its scarcity with the dominance of rheumatic valve pathology [9].

Similarly, Sir William Osler’s 1892 textbook, The Principles and Practice of Medicine, devoted fewer than three pages to angina and did not delineate MI as a distinct nosological entity [10]. Sudden cardiac deaths were vaguely categorized as “cardiac paralysis,” and many believed that complete coronary occlusion resulted in instantaneous death, a view reinforced by the absence of clinically observed survivors [11]. It was not until James Herrick’s 1912 report, linking electrocardiographic ST-segment shifts to post-mortem transmural scarring, that survivable infarction gained acceptance [12].

Autopsy evidence further illustrates CHD’s rarity. Civil War-era necropsies (1861-1865) revealed minimal coronary artery blockages, with few signs of lipid-laden plaques, despite systematic examination of over 13,000 fatalities [13,14]. In contrast, Korean War casualties (mean age 22) showed intimal atheroma in 78% of hearts and >50% stenosis in 20%, highlighting the rapid emergence of atherosclerosis by the mid-20th century [15]. Although these findings may reflect accelerated atherosclerosis due to environmental or lifestyle shifts, the age difference between cohorts introduces a confounding factor and limits direct comparison to the general population. Archival data from the Metropolitan Life Insurance Company (1885-1895) listed fewer than one infarct per 1,000 adult deaths, with infectious causes like lobar pneumonia predominating [16]. Pathological society proceedings occasionally noted calcified coronary arteries in conditions such as syphilitic aortitis, but these were anatomical oddities rather than routine findings [17].

The intellectual framework of 19th-century medicine marginalized CHD [18,19]. Authoritative texts offered minimal discourse on angina, and MI lacked a nosological identity [20-22]. Physicians, lacking diagnostic tools like electrocardiography or biomarkers such as troponin, operated within a paradigm that broadly construed heart disease, focusing on symptomatic management rather than etiological precision [23]. Life expectancy, averaging 47 years in 1900, further limited the manifestation of chronic conditions like atherosclerosis, which requires decades of processes, such as collagen deposition and elastin degradation, to become clinically significant [24-26]. Vital statistics aggregated heart disease into a heterogeneous category, obscuring CHD’s negligible contribution.

This historical context explains CHD’s minor role in 19th-century mortality [27,28]. However, by 1968, age-adjusted CHD mortality surged to over 450 per 100,000 Americans, a threefold increase from 140 per 100,000 in 1900 [29,30]. This escalation cannot be attributed solely to improved detection but rather reflects dramatic shifts in population aging [31], mass cigarette consumption [32], refined-carbohydrate [33] and hydrogenated-seed-oil diets [34], and emerging hypertension epidemics [35], all of which underscore the impact of evolving lifestyles and medical advancements on cardiovascular health [36,37].

## Review

Emergence of processed seed oils and excess linoleic acid (LA)

Linoleic acid (LA) has long been regarded as an essential and cardioprotective nutrient. This reputation stems largely from its well-established capacity to reduce serum cholesterol levels [38-41]. Numerous prospective cohort studies and meta-analyses have consistently associated higher dietary LA intake, typically ranging from 5% to 10% of total energy, with reduced risk of CHD events and mortality [42-44]. These large-scale studies provide important evidence of LA’s beneficial associations at typical intake levels. Additionally, the essentiality of LA is underscored by Barth syndrome, a rare genetic disorder in which impaired LA incorporation into cardiolipin results in fatal cardiomyopathy, often by the third decade of life [45].

However, this prevailing view may be incomplete, as it often fails to consider the potential for excessive LA to undergo peroxidation. This process can generate reactive aldehydes capable of damaging mitochondrial proteins and DNA through mechanisms such as adduct formation and covalent binding of these aldehydes to biomolecules, which may contribute to mitochondrial dysfunction. These effects, though underrecognized in conventional dietary models, may have direct relevance to coronary heart disease. In this context, adopting a balanced and comprehensive perspective becomes essential, and this manuscript aims to clarify the complex role of LA and its possible implications for both the development and prevention of CHD.

The late 19th century marked the beginning of a major shift in dietary fats, driven by the rise of industrially processed seed oils, a category that includes chemically refined vegetable oils such as cottonseed, soybean, and corn oils, as well as later hydrogenated derivatives [46]. By the 1880s, advancements in industrial refining techniques, such as alkali treatment and steam deodorization, altered their chemical profile, rendering them palatable and stable for culinary applications [47]. In the 1870s and 1880s, manufacturers blended cottonseed oil with lard, a staple saturated fat, yielding “compound” shortenings that undercut the cost of pure animal-derived lipids [48,49].

Cottonseed oil production scaled rapidly following the consolidation of the sector in 1884, when a centralized entity emerged to streamline extraction, refining, and distribution [50]. By 1887, products composed of approximately 80% cottonseed oil and 20% beef tallow had entered the market, packaged to mimic traditional shortenings [51,52]. Concurrently, the rise of margarine, typically made from partially hydrogenated vegetable oils, further entrenched seed oils in the food system [53]. By the 1880s, its popularity burgeoned, despite legislative efforts in 1886 to impose restrictive measures such as taxation, aimed at protecting the dairy industry from economic competition [54]. These regulatory challenges notwithstanding, margarine’s accessibility and versatility drove its uptake, amplifying the presence of seed oil-derived fatty acids in daily consumption.

In 1911, the first fully hydrogenated cottonseed oil shortening was introduced, a product that revolutionized food processing [55]. Through hydrogenation, a process that saturates carbon-carbon double bonds, the oil’s liquid state was transformed into a solid, enhancing its oxidative stability and shelf life [56]. This innovation broadened its culinary utility, from frying to pastry-making, and spurred rapid adoption, as evidenced by the dramatic increase in its market presence within five years of its introduction [57]. The shift to hydrogenated forms reflected the increasing use of chemical processing to modify lipid structures and aligned with a growing consumer preference for processed goods perceived as modern and efficient [58]. Consequently, per capita intake of vegetable oils and shortenings surged by the early 20th century. Early promotional efforts framed these oils as progressive alternatives, emphasizing their uniformity and cost-effectiveness and facilitating their seamless integration into both domestic and commercial food preparation [59,60].

Dietary changes and heart disease trends (1900-1930)

Historically, in 1900, CHD ranked as the fourth leading cause of death in the United States, with a mortality rate of approximately 137 per 100,000 population, significantly lower than infectious diseases [61]. Initial increases in CHD-related mortality were noted in the 1910s, with a more pronounced surge in the 1920s [62]. By 1930, death rates from CHD exceeded 200 per 100,000, marking a notable rise from the 1900 baseline. This upward trajectory persisted, reaching over 450 per 100,000 by 1968 [63], more than a threefold increase from the beginning of the century. Specifically, the age-adjusted CHD mortality among U.S. adults aged 35 years and older had soared to approximately 1,034 per 100,000. When fatal stroke is included, the aggregate cardiovascular disease (CVD) mortality for this demographic reached approximately 1,350 per 100,000, reflecting the growing burden of chronic vascular diseases during a period of shifting lifestyle, diagnostic, and dietary patterns.

Concurrently with these mortality trends, significant changes were occurring in the American diet, particularly in the types of fats consumed. Per capita use of industrially refined seed oils continued to rise through the mid-20th century, propelled by the postwar expansion of processed foods and the mainstreaming of plant-derived oils as replacements for traditional animal fats [64]. Between 1940 and 1960, domestic soybean oil production surged by more than 1000%, driven by advancements in agricultural mechanization and shifting dietary trends toward plant-based lipids.

In contrast, the consumption of traditional animal-derived fats, such as butter and lard, exhibited only modest fluctuations, typically less than a 10% change, during the same period [65]. The concurrent rise in LA-rich oils and CHD mortality suggests a possible temporal association, though such correlations cannot by themselves establish causality [66,67]. Notably, the dietary transition toward seed oils preceded the escalation in CHD mortality by an estimated 10 to 20 years, a lag interval that aligns with the known timeline of atherosclerotic progression [68]. While this alignment is suggestive, it must be interpreted within the context of other dietary and lifestyle shifts occurring during this era.

The pathogenesis of atherosclerosis hinges on a complex interplay of lipid metabolism, oxidative stress, and inflammatory cascades [69]. This chronic process, driven by factors such as lipoprotein accumulation and endothelial dysfunction, can span years or decades before precipitating symptomatic outcomes like MI [70]. Dietary fats can exert influence through their modulation of lipoprotein profiles and inflammatory pathways [71].

Peroxide LA has been shown to accelerate the oxidation of low-density lipoprotein (LDL) particles, yielding ox-LDL that contributes to endothelial dysfunction and plaque formation, a key step in atherogenesis [72,73]. The balance of omega-6 to omega-3 polyunsaturated fatty acids (PUFAs) may shape systemic inflammatory responses [74]. Seed oils, rich in LA, altered the fatty acid composition of the American diet, likely tilting the inflammatory equilibrium toward a pro-atherogenic state. Although their significance was not fully appreciated during the early phases of dietary industrialization, the presence of trans fats may have compounded the atherogenic potential of seed oils, particularly as total fat consumption rose [75].

Fat consumption in the early 20th century emphasized saturated fats from butter and lard, which differ markedly in their metabolic effects from the LA predominant in seed oils. The transition to seed oils coincided with a doubling of heart disease rates between 1900 and 1930, a phenomenon rendered more striking by the concurrent decline in overall mortality due to advancements in sanitation and medical care [76]. This paradox highlights the potential role of dietary change as a contributing factor in the emergence of cardiovascular disease.

Moreover, the temporal association between seed oil adoption and CHD extends beyond a single population. Regions integrating processed oils into their diets at later intervals often exhibited corresponding delays in heart disease mortality, reinforcing the hypothesis that this dietary shift may contribute to atherogenic processes [77,78]. By the mid-20th century, evidence of coronary atherosclerosis in younger individuals suggested that vascular pathology was occurring earlier in life, potentially reflecting the cumulative impact of prolonged exposure to these dietary patterns, an outcome consistent with the chronic nature of plaque development [79].

Mid-century inflection and persistent CHD trends (1960-present)

Consequently, the period from 1900 to 1930 encapsulates a seminal period in nutritional epidemiology, where the escalation of seed oil consumption paralleled an unprecedented rise in heart disease [80,81]. The increased intake of total fats, coupled with the specific properties of processed oils, plausibly amplified the burden of atherosclerosis [82]. This dietary evolution, set against a backdrop of stable or diminishing intake of other macronutrients, raises the possibility that seed oils may have acted as a modulator of cardiovascular trends, their effects unfolding over decades as arterial integrity gradually eroded [83,84]. 

**Figure 1:**
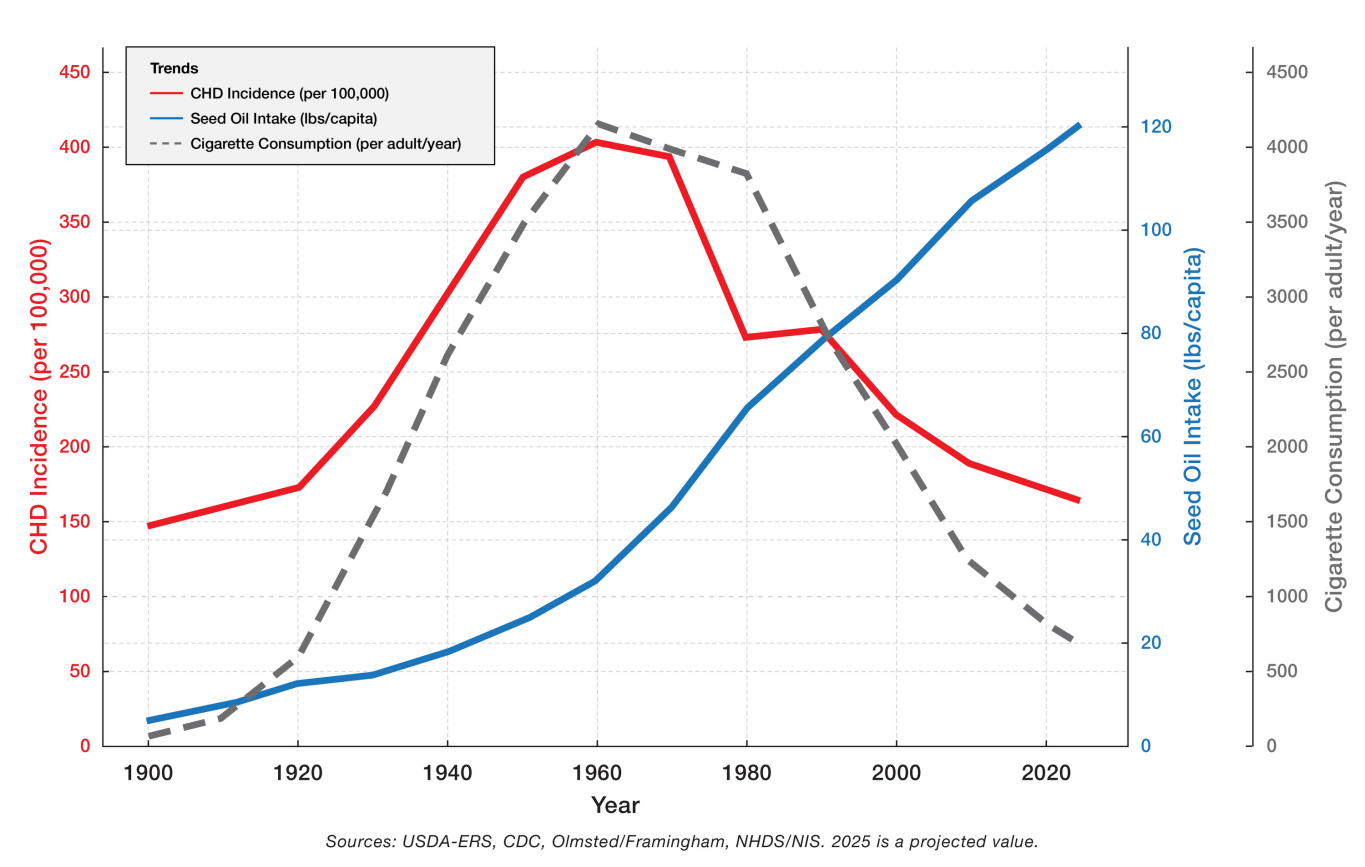
Temporal trends in U.S. seed oil intake, cigarette consumption, and CHD incidence from 1900 to 2025. The solid red line represents CHD incidence (per 100,000 individuals), which increased from approximately 150 in 1900 to a peak of around 400 in the 1960s, then declined to about 150 by 2020. The solid blue line depicts seed oil intake (lbs/capita), rising steadily from near zero in 1900 to a projected 120 lbs/capita by 2025, with a marked post- 1940 acceleration coinciding with the widespread adoption of industrial oils such as soybean oil. The dashed gray line illustrates cigarette consumption (cigarettes per adult/year), which increased from 500 in 1900 to approximately 4,000 in the 1960s, then fell to 1,000 by 2020. Historically, dietary patterns and tobacco use have been prioritized as key risk factors for cardiovascular disease, and the data suggest a possible mid-century correlation between increasing seed oil intake and CHD incidence. Note: This figure represents aggregated, ecological data intended for hypothesis generation. Image Credit: Joseph Mercola, DO

Figure 1 illustrates the overlapping rise and fall of seed oil intake, cigarette use, and CHD incidence in the U.S. from 1900 to 2025. The temporal alignment of these trends underscores a possible mid-century inflection point in CHD risk, coinciding with a sharp increase in LA-rich oils. Although these trends are temporally aligned, their aggregation does not imply causation. Numerous confounding factors, including socioeconomic changes, evolving diagnostic practices, and concurrent public health campaigns, complicate interpretation.

According to USDA Economic Research Service data and food-availability records, U.S. per capita consumption of vegetable oils surged from 4.5 kg in 1909 to 45.4 kg by 1970, with a total supply increase of approximately 160% by 2010 [85], surpassing the physiological threshold of 2-3% where tissue phospholipid LA saturation plateaus [86]. In contrast, cigarette consumption surged from 54 cigarettes per adult in 1900 to 4,345 by 1963, an 80-fold increase [87], introducing millimolar concentrations of reactive oxygen species (ROS), such as 0.1-0.5 mM hydrogen peroxide per cigarette, into an arterial environment enriched with LA-laden LDL particles [88]. Following the 1964 Surgeon General’s report, smoking prevalence declined modestly from 42% to 38% between 1968 and 1976, yet CHD mortality decreased by 20.7% during this period, suggesting that factors beyond smoking were influential [89]. Indeed, IMPACT modeling estimates that only 12.5-25% of the CHD mortality decline from 1980 to 2000 can be attributed to smoking cessation [90].

USDA food-availability records evaluated by multiple research teams reveal a marked rise in vegetable oil supply, particularly LA-rich oils. Specifically, the total vegetable-oil supply increased by approximately 160% between 1909 and 2010, reaching an estimated 45 kg per capita by 2010 [91]. Loss-adjusted data series and nutrient composition tables from the National Health and Nutrition Examination Survey (NHANES) indicate that reformulations in the 1970s shifted the fatty acid profiles of salad and cooking oils towards high-oleic, monounsaturated fatty acid (MUFA)-rich variants, a change driven by emerging evidence linking dietary fat composition to cardiovascular health [92,93].

Campaigns in the late 1970s aimed at reducing caloric intake and promoting lower-fat diets preceded a notable decline in mean LA consumption, which fell to approximately 10-11 grams per day by the mid-1980s [94,95]. This reduction proved transient; however, as LA intake rebounded during the 1990s in response to shifting dietary patterns and food industry practices. Although achieving precise gram-for-gram alignment across datasets remains challenging, owing to inconsistencies in accounting for food waste and under-reporting, the collective evidence from these studies consistently substantiates the numerical trends described.

This decline preceded and paralleled a 30% reduction in CHD incidence (550 to 385 cases per 100,000) between 1970 and 1985, whereas smoking prevalence decreased more gradually (42% to 33%) [96-99]. The transient reduction in LA intake during the late 1970s and early 1980s, driven by dietary campaigns and reformulations, may have played a contributing role in the observed 30% decrease in CHD incidence from 1970 to 1985, as lower LA levels would reduce the substrate for oxidative modification and inflammatory signaling in the arterial wall.

Pharmacotherapeutic advancements in the 1970s [100], including coronary care units reducing MI case fatality by 20% and aspirin lowering recurrent MI by 24%, contributed significantly to mortality declines; however, their impact was primarily therapeutic, not preventive [101-103]. From a mechanistic standpoint, LDL particles enriched with LA exhibit heightened susceptibility to oxidative modification [104]. The interplay between oxidative triggers and lipid substrates offers one possible framework for interpreting epidemiological trends. CHD mortality declined significantly after the 1960s, a period that saw reductions in smoking-related oxidative stress. However, dietary intake of PUFAs, particularly LA, remained persistently elevated, continuing to provide a lipid substrate potentially vulnerable to peroxidation within the vascular environment [105,106].

This mechanistic interplay between oxidative stressors and oxidizable lipoproteins could help explain why CHD incidence has remained above pre-industrial baselines, historically estimated at <100 cases per 100,000 persons annually, despite reductions in key external triggers. Even with advancements in public health and medicine, the persistent elevation of LA intake sustains a higher baseline of oxidative potential, thereby maintaining CHD incidence above pre-industrial levels and possibly contributing to the emergence of other LA-sensitive conditions. Furthermore, it may contribute to the rising prevalence of LA-sensitive cardiometabolic conditions, such as metabolic dysfunction-associated steatotic liver disease (MASLD), heart failure with preserved ejection fraction (HFpEF), and insulin resistance [107-109].

In this context, LA functions not as a sole etiological factor but as a permissive modulator of allostatic load, the cumulative physiological burden from chronic stress adaptation [110]. By potentially amplifying vascular damage from intermittent oxidative insults, such as those from residual smoking or air pollution, elevated LA may sustain a pro-inflammatory and pro-atherogenic state [111,112].

Cigarette additives as catalysts in the cardiovascular disease epidemic

The cardiovascular impact of cigarette smoking was not solely attributable to the combustion of tobacco itself. By the 1940s and 1950s, manufacturers began incorporating a suite of chemical additives into cigarettes, including humectants such as glycerol, preservatives like sorbic acid, flavorants such as licorice extract, and pH modifiers like diammonium phosphate [113-116].

These compounds, originally intended to enhance shelf life and palatability, substantially modified the chemical profile of cigarette smoke [117]. Upon pyrolysis, these additives decompose to yield reactive oxygen species (ROS), notably superoxide anions (O₂•⁻), hydrogen peroxide (H₂O₂), hydroxyl radicals (•OH), and peroxyl radicals (ROO•) [118]. Glycerol, for instance, thermally degrades into acrolein, a reactive aldehyde present in concentrations up to 150 μg per cigarette, which intensifies ROS generation via redox cycling [119].

Concurrently, dietary patterns began to shift. The widespread adoption of processed seed oils rich in LA increased their accumulation in tissues such as the vascular endothelium and in alveolar surfactant layers [120]. In this context, ROS from cigarette smoke may initiate lipid peroxidation by abstracting bis-allylic hydrogens from LA molecules [121]. This reaction produces lipid hydroperoxides, which degrade into cytotoxic aldehydes, including 4-hydroxynonenal (4-HNE) and malondialdehyde (MDA). Plasma 4-HNE levels in smokers have been measured in the micromolar range. These byproducts can disrupt membrane integrity, oxidize LDL, and promote endothelial inflammation, processes implicated in atherogenesis [122].

Importantly, the downstream effects of this oxidative cascade appear to impact multiple hallmarks of cardiovascular pathology [123]. Lipid peroxidation byproducts may impair antioxidant defenses by inactivating enzymes such as superoxide dismutase (SOD), with activity reductions of up to 30% observed in chronic smokers, while also promoting foam cell formation, a key step in atherosclerotic plaque development [124-126]. These synergistic insults compound vascular injury over time. Framed within the broader hypothesis of this paper, the convergence of high dietary LA and cigarette additive-induced oxidative stress offers a plausible mechanism by which the vascular system faced both an abundance of oxidizable substrates and a sustained oxidative trigger during the mid-20th-century rise in CHD [127].

In light of this proposed synergy, mitigating cardiovascular risk may require a more integrated strategy. Smoking cessation remains paramount, yet reducing dietary intake of processed seed oils could help limit the oxidative substrate pool burdening vascular tissues [128]. While public health policy has traditionally focused on tobacco control, dietary interventions may complement these efforts and strengthen outcomes in the ongoing fight against cardiovascular disease.

Alternative factors in the rise of heart disease

In parallel, refined sugar consumption also increased steadily during this period, transitioning from a modest intake to a more substantial presence in diets by the 1920s. This gradual rise, however, stands in contrast to the abrupt surge in CHD rates, suggesting that sugar’s role may have been ancillary rather than primary. Excessive sugar intake promotes insulin resistance and elevates triglycerides, indirectly exacerbating atherogenesis [129]. In this context, more acutely impactful factors, such as smoking or seed oils, likely played a larger role in triggering the rapid early uptick in CHD.

Advancements in diagnostic capabilities further influenced the apparent rise in CHD prevalence [130]. The electrocardiogram (ECG) in the 1910s revolutionized the identification of MI by detecting ischemic alterations in cardiac electrical activity, such as ST-segment elevation, enabling clinicians to diagnose conditions that might have previously gone unrecognized [131,132]. This enhanced diagnostic potential likely contributed to an increase in reported CHD cases, reflecting not only a genuine rise in incidence, as indicated by the aforementioned autopsy data from Korean War soldiers, but also a refinement in detection methods [133].

Similarly, refinements in nosological frameworks, such as the implementation of the International Classification of Diseases (ICD) coding by national health agencies, enhanced the precision of CHD documentation [134]. Standardized classification systems mitigate diagnostic ambiguity by defining specific cardiovascular pathologies, such as angina pectoris or coronary occlusion, thus reducing the likelihood of misattribution to nonspecific causes [135]. For instance, ICD-4 reduced vague diagnoses by an estimated 15%, which likely amplified the recorded prevalence of heart disease. This suggests that part of the observed increase may stem from improved disease categorization rather than an exclusive escalation in underlying pathology [136].

Demographic transitions, notably population aging, also merit consideration as contributing factors [137]. As life expectancy extended from approximately 47 years in 1900 to 60 years by 1930, a larger proportion of individuals entered age strata where CHD prevalence naturally peaks [138,139]. However, even when age-adjusted rates are considered, accounting for the proportional burden across age groups, CHD mortality continued its upward trajectory. This persistence suggests that aging is a likely confounding factor that propelled the epidemic beyond mere demographic predictability.

Additionally, the societal shift toward urbanization and diminished physical activity likely exacerbated CHD susceptibility [140]. Shifting from physically demanding agrarian lifestyles to sedentary urban occupations reduced energy expenditure, contributing to conditions such as visceral adiposity and hypertension, both precursors to atherosclerosis. Environmental stressors linked to urban living, including poor air quality and psychosocial strain, have been implicated in amplifying cardiovascular risk. Moreover, dietary shifts accompanying urbanization have been associated with increased CVD risk [141]. While these lifestyle alterations undoubtedly shaped disease trends, their relative weight amid concurrent factors remains challenging to isolate.

Despite the multiplicity of these influences, the dietary incorporation of seed oils emerges as a noteworthy temporal correlate with the rise in CHD [142]. While peak smoking rates in the 1950s and 1960s contributed to a continued rise in CHD, the introduction of PUFAs through processed vegetable oils marked a simultaneous change in the American dietary fat profile. This nutritional departure altered lipid metabolism in ways that potentially predisposed to atherosclerosis via pathways such as increased LDL oxidation [143]. Although other contributors were at play, the hypothetical role of seed oils is emphasized here due to both the mechanistic plausibility and temporal alignment with early CHD escalation [144].

Although cigarette smoking, refined sugar, diagnostic advancements, nosological refinements, aging, and societal shifts likely contributed to the early 20th-century CHD epidemic, the impact of the dietary introduction of processed seed oils should not be overlooked [145]. While smoking and lifestyle changes likely amplified an existing trend, sugar’s effects were indirect, and diagnostic and reporting improvements reflected rather than caused the rise [146]. Aging and urbanization may have shaped overall disease risk, but do not fully explain the timing or magnitude of early 20th-century trends. In contrast, the rapid, widespread adoption of LA-rich oils, which tripled dietary LA intake within decades, corresponds with the initial inflection point in CHD incidence, supporting its role as a hypothetical central driver.

Taken together, these data suggest that dietary LA contributes significantly to the residual burden of CHD, even after substantial mortality declines since 1980 [147]. Despite a 60% reduction in U.S. age-adjusted CHD mortality to approximately 150 per 100,000, rates remain at least 50% higher than pre-industrial estimates of less than 100 per 100,000 [148]. Using the IMPACT model diet-risk coefficients and re-analyses of trials like the Minnesota Coronary Experiment (MCE) and Sydney Diet Heart Study (SDHS), which found no mortality benefit from replacing saturated fatty acids with high-LA corn oil, it is estimated that reducing mean LA intake from the current 6-8% of total energy (%E) to a historical 3% could decrease oxLDL levels by approximately 0.15 standard deviations [149]. These findings provide large-scale epidemiological support for a potential adverse impact of excess dietary LA [150].

Within the contemporary English IMPACT framework, this shift corresponds to a 9-18% further reduction in CHD mortality, equivalent to approximately 45,000 fewer annual U.S. deaths (range 25,000-65,000) after a five-year lag [151]. This projection mirrors the 14% decline in ischemic heart disease mortality observed in Denmark following its 2004 industrial trans-fat ban, which reduced dietary trans-fatty acids to less than 1% of energy intake [152]. This is presented not as a mechanistic comparison between trans fats and LA, but as an illustrative example of the impact of policy-driven dietary change on CHD outcomes. These findings underscore the potential for targeted dietary interventions to mitigate persistent CHD risk, reinforcing the need to reevaluate LA-rich seed oils in modern nutritional guidelines.

Mechanistic links and biological plausibility

Seed oils are highly susceptible to oxidation due to their high LA content [153]. This reactivity enables PUFAs to form lipid peroxides. Consequently, these processes introduce cytotoxic aldehydes like 4-HNE into the diet. In the cardiovascular system, these oxidized lipids impair endothelial function, a key initiating factor in atherosclerosis [154-156].

**Figure 2:**
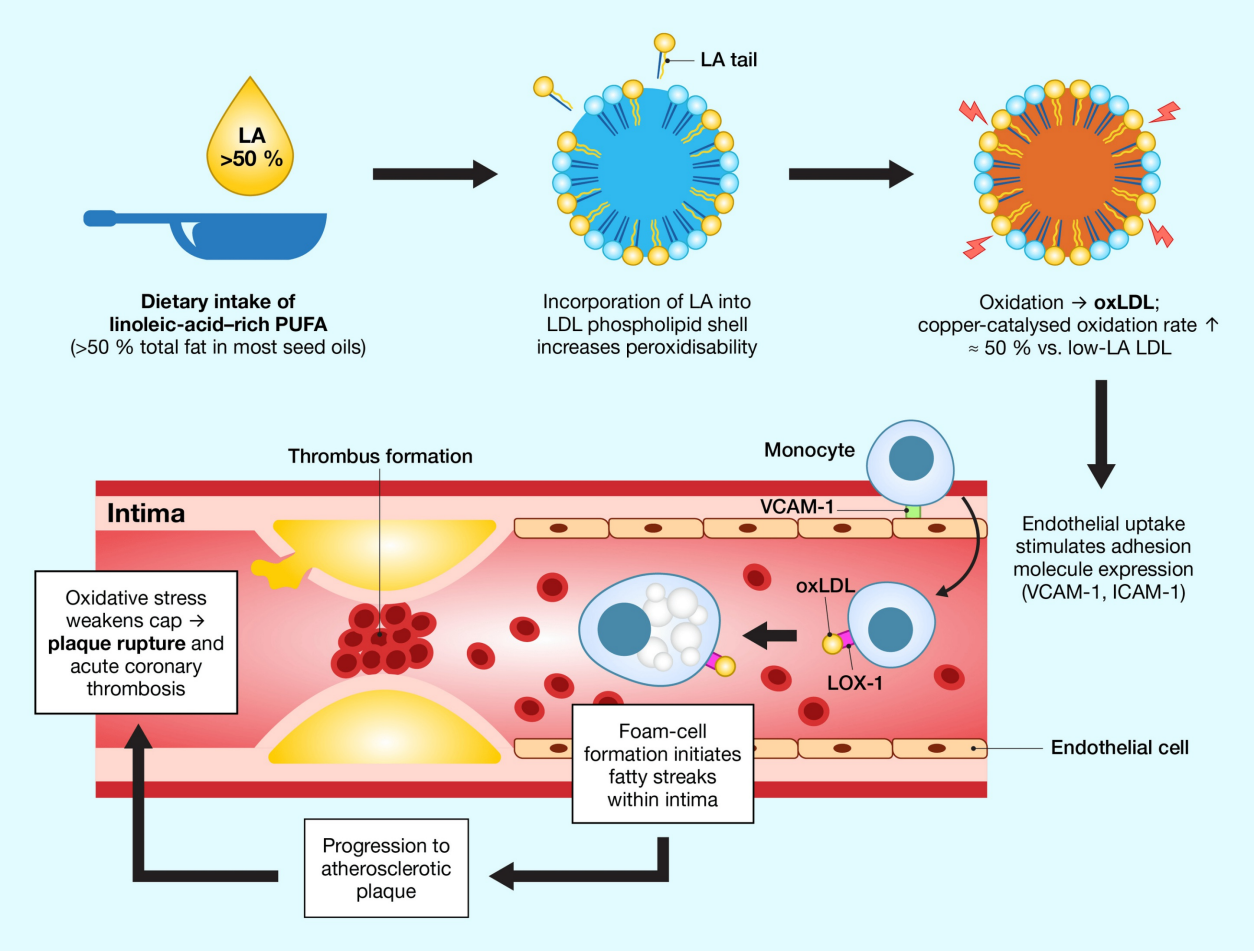
Oxidative cascade from LA-rich seed-oil ingestion to atherothrombotic plaque rupture. Dietary LA (>50% of total fatty acids in most industrial seed oils) is incorporated into the phospholipid shell of circulating LDL, which has been shown to increase LDL susceptibility to copper-mediated oxidative modification compared with more oleate-rich/low-LA LDL [160,161]. The resulting oxidized LDL (oxLDL) up-regulates endothelial adhesion molecules (VCAM-1, ICAM-1), thereby promoting monocyte recruitment. Monocytes differentiate into macrophages, which internalize oxLDL via LOX-1 and related scavenger receptors, forming cholesterol-laden foam cells that initiate fatty-streak formation within the vascular intima. Progressive lipid accumulation and sustained oxidative stress weaken the fibrous cap, predisposing the mature plaque to rupture and precipitating acute coronary thrombosis. Image Credit: Joseph Mercola, DO

As depicted in Figure 2, lipid oxidation, particularly in LDL, is a driver of atherosclerosis. Macrophages internalize oxLDL via scavenger receptors, becoming foam cells laden with lipid droplets [157]. Recent investigations demonstrate that oxLDL loading epigenetically silences AKT serine/threonine kinase 2 (AKT2)-dependent acetyl-CoA generation, thereby suppressing histone H3 lysine 27 (H3K27) acetylation at inflammatory gene loci and locking macrophages into a pro-atherogenic metabolic state. This triggers an inflammatory cascade within the arterial intima, characterized by the release of cytokines and chemokines such as interleukin-1 (IL-1) and monocyte chemoattractant protein-1 (MCP-1) [158]. As a result, atherosclerotic plaques expand, constricting the arterial lumen and increasing CHD risk [159].

Since the 1960s, research has emphasized that diets high in oxidizable fats, such as PUFAs, may promote arterial pathology [162]. Under normal conditions, the body’s endogenous antioxidant systems, including enzymes like glutathione peroxidase and catalase, mitigate oxidative stress and reduce lipid peroxidation [163]. However, experimental frameworks have long posited that the incorporation of oxidation-prone lipids into the diet may accelerate the formation of atherosclerotic lesions through heightened oxidative stress and inflammation [164]. This line of investigation helped catalyze early interest in differentiating the physiological effects of various dietary fats, a theme that has remained central in nutritional science [165]. Pathological observations further illuminate this relationship, revealing a temporal increase in coronary artery lesions that parallels the widespread adoption of processed vegetable oils [166]. Recent analysis suggests that LA-rich seed oils are associated with increased CHD risk, whereas odd-chain saturated fats (OCSFAs) like pentadecanoic acid (C15:0) show inverse associations in cohort studies [167].

In contrast, animal-derived fats, predominantly composed of saturated fatty acids, exhibit greater resistance to oxidative modification due to their lack of reactive double bonds.

The peroxidation of PUFAs yields bioactive aldehydes, such as 4-HNE and MDA, which damage proteins and nucleic acids [168]. These molecular alterations can upregulate gene expression, favoring smooth muscle cell proliferation and extracellular matrix degradation, processes that destabilize atherosclerotic plaques [169]. Thus, a plausible mechanistic link exists between dietary fat quality and coronary disease progression. However, it is important to note that PUFAs have also been associated with certain cardioprotective properties, including modulation of plasma lipids and membrane fluidity [170], though such benefits may depend on dose, context, and specific fatty acid subtype.

Collectively, these biological mechanisms, LA oxidation [171], trans fat generation [172], and lipid peroxidation [173], offer a mechanistically coherent model for the observed surge in coronary atherosclerosis by the 1930s [174]. The widespread adoption of seed oils, a departure from traditional fats, introduced a novel potentiator of CHD risk [175]. This dietary evolution, rooted in the biochemical properties of processed fats, plausibly contributed to the escalating incidence of CHD. These trends are consistent with a complex interplay between nutrition and cardiovascular health.

Linking seed oils to the coronary heart disease epidemic

The putative link between LA-rich seed oils and the 20th-century CHD epidemic can be evaluated using epidemiological criteria for causation, such as the Bradford Hill criteria [176]. These criteria, temporality, strength of association, consistency, biological gradient, plausibility, experiment, coherence, specificity, and analogy, were historically developed to advance scientific reasoning about causality. They provide a structured framework to determine whether the rapid, population-wide adoption of LA-rich fats plausibly contributed to the rise in CHD incidence [177]. In this context, the framework is particularly suitable for examining the hypothesis that increased LA consumption may have played a role in the CHD epidemic. This section applies each criterion in turn to develop a structured hypothesis regarding possible causality [178].

Temporality

A cornerstone of causal inference, temporality requires that exposure precedes the outcome. In this context, the advent of industrial seed oils ushered in elevated LA consumption beginning in the late 19th century; per capita supply in the United States rose from <0.5 kg (1900) to ≈11 kg (1960) [179]. This dietary transition, driven by innovations in food production, altered the fatty acid profile of populations, particularly in urbanized settings [180]. Subsequently, the early to mid-20th century saw a marked increase in CHD, with conditions like atherosclerosis manifesting more prominently from the 1920s onward. The interval between these events, spanning approximately 10 to 20 years, aligns with the chronic progression of cardiovascular pathology, where lipid accumulation in arterial walls develops gradually [181]. This lag is consistent with the hypothesis that increased LA intake could initiate or exacerbate such processes. For instance, consumption of animal-derived fats, rich in saturated fatty acids, remained stable or elevated historically without a proportional rise in CHD, suggesting that the type of fatty acid, rather than total fat intake, may be the key [182].

Strength of Association

**Figure 3:**
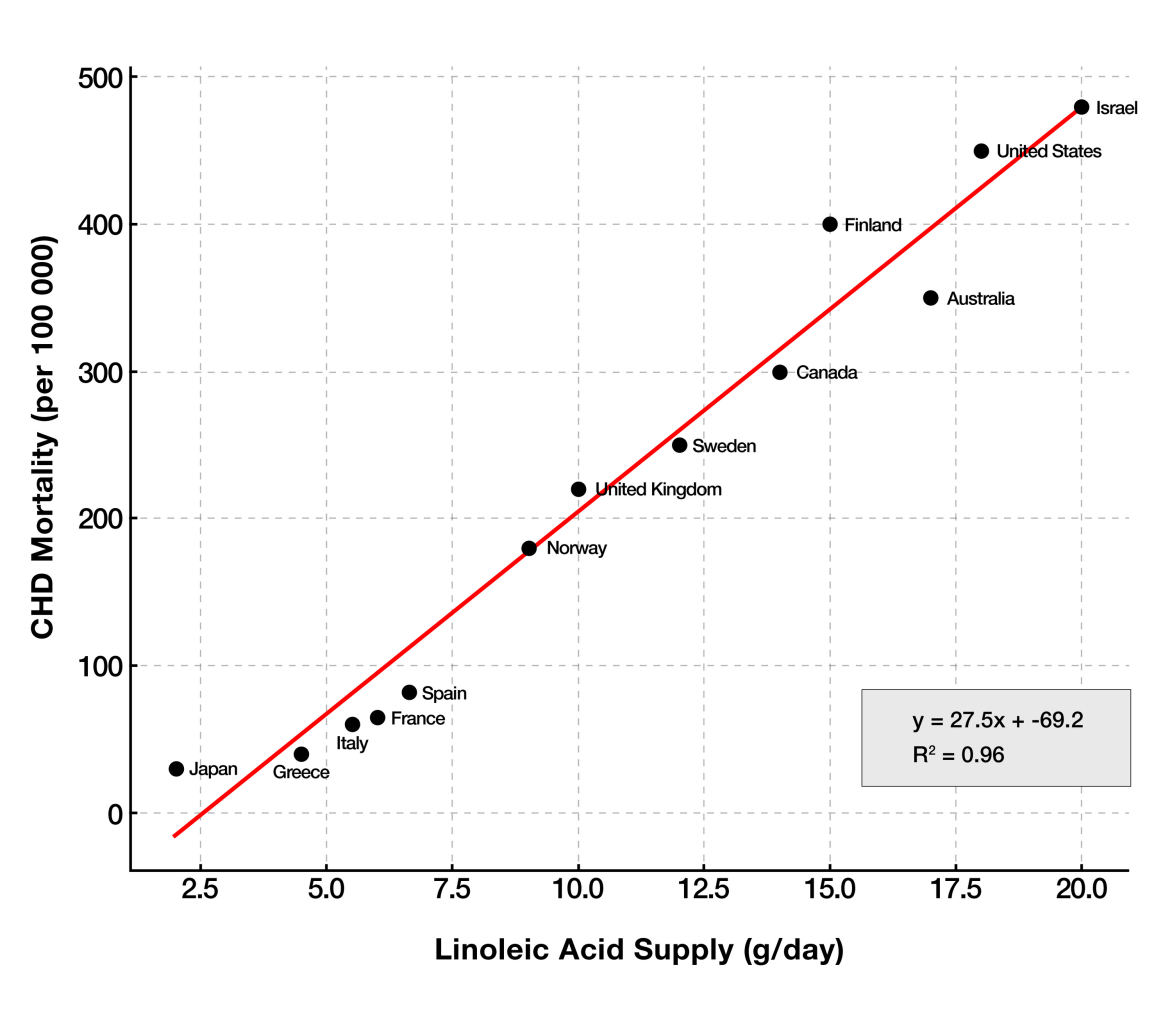
Cross-national correlation between linoleic acid supply and CDH mortality. Each point denotes a country; the fitted least-squares line (β=27.5 deaths/100,000 per g LA/day, R²=0.96, p<0.001) reveals a strong positive gradient. Nations with the greatest LA availability—Israel (~20 g/day) and the United States (~18 g/day)—experienced the highest CHD mortality (>450 deaths/100,000), whereas countries retaining traditional low-LA diets such as Japan and Greece (<3 g/day) exhibited markedly lower rates (<50 deaths/100,000). The uniform dose–response pattern, despite heterogeneous genetics and lifestyles, aligns with the hypothesis that chronic LA excess amplifies atherogenic risk at the population level. Due to the ecological nature of this analysis and lack of adjustment for confounding factors (e.g., smoking, hypertension, diet), this figure is presented as supportive but not determinative. Image Credit: Joseph Mercola, DO

As depicted in Figure 3, regions that integrated LA-rich seed oils, the earliest industrialized U.S. cities and Northern Europe, recorded CHD mortalities >800 per 100,000 by the 1960s, whereas Mediterranean locales relying on MUFA-rich olive oil remained <200 per 100,000 [183]. This geographic gradient highlights a pattern in which increasing LA exposure correlates with elevated disease burden [184,185]. Furthermore, a possible dose-response relationship emerges as LA rises from ≈2% to >7% of total energy (Figure 3), CHD risk and mortality escalate in parallel. However, this ecological pattern is vulnerable to confounding and does not establish causality. Many factors, including smoking rates, diagnostic criteria, and healthcare access, differ across countries. While there is a suggestion that deeper incorporation of LA into cell membranes and lipoproteins amplifies cardiovascular risk [186], these associations should be interpreted as hypothesis-generating rather than definitive.

Consistency

Societies adopting seed oils, particularly in industrialized Western settings, experienced uniform CHD increases, whereas populations retaining low-LA, traditional fats showed markedly lower rates [187]. Temporal consistency is also evident: in the United States, partial replacement of hydrogenated seed oils with lower-LA alternatives during the 1980s coincided with an initial downturn in CHD mortality, preceding modern pharmacotherapy [188,189]. However, CHD rates also declined while LA intake remained high, which complicates a simple interpretation. Although multifactorial influences blur absolute attribution, repeated observation across demographics may support causal inference and align with LA’s pro-inflammatory and pro-oxidative biochemical profile [190-192]. In epidemiological terms, such recurrence across settings is consistent with a potential causal mechanism, possibly tied to LA’s role in inflammatory pathways or lipid peroxidation, which could potentially predispose individuals to cardiovascular damage over time.

Biological Gradient

The concept of a biological gradient, or dose-response relationship, serves as a foundational element in assessing causality. This principle asserts that increased exposure to a potential risk factor should correspond to a proportional rise in effect, such as the incidence or severity of a disease. In examining the link between LA and CHD, a theoretical framework emerges from the historical rise in LA consumption [193]. As dietary fats shifted from traditional animal sources to industrial seed oils during the 20th century, LA intake climbed sharply, mirroring the concurrent increase in CHD prevalence [194,195]. However, the CHD decline post-1960s despite continued high LA intake suggests a more complex relationship and highlights the potential influence of other factors.

Plausibility and Coherence

These elements further deepen this exploration of possible causality by requiring a biologically feasible mechanism for how LA intake might contribute to CHD. Excessive LA can elevate systemic oxidative stress, generating oxLDL-the proximate driver of foam-cell formation [196]. LA enriches LDL phospholipids, increasing their susceptibility to copper-induced oxidation by ≈50% in vitro. This could elevate systemic inflammation or oxidative stress, processes widely recognized as central to the pathogenesis of atherosclerosis [197,160,161]. This dietary shift, marked by the displacement of low-LA fats with LA-rich seed oils, contrasts with pre-industrial eating patterns, where minimal LA exposure coincided with lower CHD rates. Taken together, these patterns suggest a dose-dependent relationship in which greater dietary LA may align with heightened cardiovascular risk [198]. Nonetheless, it should be considered that mechanistic findings derived from in vitro models may not fully capture in vivo dynamics.

Experiment (Natural/Quasi-Experimental Evidence)

Conducting controlled, population-wide trials is often impractical due to logistical and ethical constraints; however, natural experiments provide valuable insights into public health phenomena. During the 1940s, wartime rationing across Europe significantly reduced the importation of seed oils. Contemporaneously, CHD mortality rates decreased markedly in occupied Norway [199], where annual CHD deaths fell by approximately 30%, as well as England and Wales [200]. Upon the restoration of trade following the war, per-capita LA availability surged, followed by increased CHD incidence. This trend was first documented in Norway and later observed in Finland’s North Karelia cohort, where CHD mortality increased by nearly 20% within a decade [201,202]. While these data suggest a temporal link, the events were accompanied by other lifestyle changes, so causation cannot be isolated.

Concurrently, data from the United States food supply reveal that LA intake escalated from less than 3% to over 7% of total energy intake during the same period [203]. Furthermore, community-level interventions that replaced partially hydrogenated shortenings, rich in trans fatty acids, with high-oleic oils have demonstrated significant vascular benefits. Specifically, randomized crossover trials have reported improvements in brachial flow-mediated dilation, a critical indicator of endothelial function, after 4 to 6 weeks [204-206]. While these findings suggest that endothelial dysfunction associated with excessive trans-fat intake is at least partially reversible [207], the studies primarily compare MUFAs with trans fats, not LA with no, LA diets-making it difficult to isolate LA-specific effects.

Analogy and Specificity

The temporal relationship between LA consumption and CHD incidence parallels the well-established smoking-lung cancer paradigm, which is characterized by the introduction of a novel exposure, a latency period spanning decades, and the subsequent emergence of epidemic disease [208]. Similar to cigarette smoke delivering persistent oxidants to pulmonary tissue, seed oils enriched with LA supply highly peroxidizable fatty acids, such as 9- and 13-hydroxyoctadecadienoic acid (HODE), that accumulate within LDL particles and the arterial intima.

Diets high in LA increase LDL’s susceptibility to oxidative stress, elevating copper-induced oxidation by 40-50% in some in vivo experimental models [160] [161]. This oxidative process generates bioactive lipid mediators like 9- and 13-HODE, which activate peroxisome proliferator-activated receptor gamma (PPAR-γ) in macrophages and promote foam-cell formation, an early hallmark of atherosclerosis [209]. This pathway could represent a plausible mechanistic explanation for heightened LA consumption as a significant contributor to CHD pathogenesis [210]. However, PPAR-γ activation is context-dependent and not exclusively pro-atherogenic. While the analogy is compelling, the LA-CHD link remains under investigation and is not yet considered established.

Recovered data from the Minnesota Coronary Experiment (MCE) and Sydney Diet Heart Study (SDHS) challenge the long-held belief that replacing even-chain saturated fatty acids with high-LA vegetable oils consistently benefits cardiovascular health. In the MCE, patients swapping butter for corn oil reduced serum cholesterol by 14%, yet mortality increased in those under 65 [211]. Similarly, the SDHS found worsened coronary mortality with LA-rich oil substitution [150]. Updated meta-analyses of similar trials have shown mixed outcomes, with no clear consensus on mortality benefit or harm [212-214]. 

In this context, this analysis provides theoretical support for a possible causal relationship between dietary LA and CHD. Temporality, strength of association, consistency, biological gradient, plausibility, experimental evidence, and analogy are addressed across multiple lines of evidence, with cross-sectional and historical data suggesting that LA intake may contribute to CHD risk. Specificity and coherence are less emphasized, but their absence is acceptable for multifactorial diseases like CHD. While correlation does not prove causation, the alignment across multiple criteria supports further investigation. The evidence suggests that LA consumption from processed seed oils could have contributed to the CHD epidemic, though confirmation would require more targeted empirical data.

## Conclusions

This historical analysis explores the potential role of processed seed oils in the early 20th-century rise of CHD. In the 1850-1900 period, CHD was rare, with autopsies showing minimal coronary atherosclerosis, while infectious diseases dominated mortality. By 1968, CHD mortality had surged to over 450 per 100,000, a threefold increase from 1900 levels, coinciding with the widespread and sustained adoption of seed oils such as cottonseed oil, introduced via products like Crisco in 1911 and further entrenched in diets through the mid-20th century. Temporally, the increase in LA-rich oil consumption preceded this surge by decades, aligning with the chronic nature of atherosclerotic plaque formation. Mechanistically, the oxidation of PUFAs into lipid peroxides and pro-inflammatory eicosanoids presents a biologically plausible pathway.

Although factors such as smoking (which rose in the 1920s) and diagnostic advancements like the ECG undoubtedly influenced CHD trends, their timing and relative impact suggest a contributory rather than primary role. This paper outlines how industrial dietary shifts may have transformed cardiovascular epidemiology and encourages a reevaluation of dietary fat sources. While prospective cohort data and controlled trials offer mixed findings regarding LA intake and CHD risk, historical and mechanistic considerations raise important questions. As such, this analysis proposes that the potential benefits of a dietary shift away from processed seed oils in favor of traditional fats warrant further investigation. Policy experiments, such as Denmark’s ban on industrial trans fats, which correlated with a 14% reduction in CHD mortality, highlight how supply-side lipid reform may produce meaningful population-level effects. Future research should prioritize prospective studies and modeling approaches to assess the impact of dietary fat composition on cardiovascular outcomes, while acknowledging the complex and multifactorial nature of CHD.
